# Consensus Bayesian assessment of protein molecular mass from solution X-ray scattering data

**DOI:** 10.1038/s41598-018-25355-2

**Published:** 2018-05-08

**Authors:** Nelly R. Hajizadeh, Daniel Franke, Cy M. Jeffries, Dmitri I. Svergun

**Affiliations:** European Molecular Biology Laboratory (EMBL) Hamburg Outstation, DESY, Hamburg, Germany

## Abstract

Molecular mass (MM) is one of the key structural parameters obtained by small-angle X-ray scattering (SAXS) of proteins in solution and is used to assess the sample quality, oligomeric composition and to guide subsequent structural modelling. Concentration-dependent assessment of MM relies on a number of extra quantities (partial specific volume, calibrated intensity, accurate solute concentration) and often yields limited accuracy. Concentration-independent methods forgo these requirements being based on the relationship between structural parameters, scattering invariants and particle volume obtained directly from the data. Using a comparative analysis on 165,982 unique scattering profiles calculated from high-resolution protein structures, the performance of multiple concentration-independent MM determination methods was assessed. A Bayesian inference approach was developed affording an accuracy above that of the individual methods, and reports MM estimates together with a credibility interval. This Bayesian approach can be used in combination with concentration-dependent MM methods to further validate the MM of proteins in solution, or as a reliable stand-alone tool in instances where an accurate concentration estimate is not available.

## Introduction

For a structural biologist, the appeal of small-angle x-ray scattering (SAXS) relates to its ability to characterize widely diverse macromolecular systems in solution. With minimal sample preparation, SAXS can be used to extract structural parameters from folded as well as flexible or intrinsically disordered proteins ranging in molecular masses (MM) from a few kDa to MDa^1–3^. The MM is among the first parameters to be determined as it is used to assess the solution state of the sample, such as oligomerization, aggregation or degradation^4^, making it integral to the data-analysis process^5^. For example, for a monodisperse protein sample, the MM estimate can be directly related to the expected MM from the protein sequence. This feature makes the MM uniquely suited for assessing the quality of the sample, the data and for guiding the modelling procedures^1,6^.

There are multiple ways of estimating the MM of proteins from SAXS data and they can be distinguished based on whether or not the protein concentration is required for the calculation. Concentration dependent methods utilize the property that the extrapolated forward scattering *I*(0) is directly proportional to the product of the particle volume and contrast squared. Here, the *I*(0) is combined with the partial specific volume, scattering density and sample concentration^7^ to arrive at the MM estimate. Of these values, only the *I*(0) is directly obtained from the SAXS experiment, for instance from Guinier approximation^8^. In addition, the intensity must be available on an absolute scale^9^, which necessitates the use of secondary scattering standards such as pure water^10^ or glassy carbon^11^. It is also possible to use the ratio of the *I*(0) from the sample and of a protein with known MM to extract the sample MM^1,6^, thereby assuming that the partial specific volume and contrast of the two proteins are identical. However, this procedure still requires an accurate determination of the concentration, in this case of both the sample and the protein standard. This fact, together with the need for a separate measurement of a standard complicates the MM determination from concentration dependent methods, and reduces the accuracy of the MM, i.e. the degree to which the estimate differs from the actual MM, in practice to no better than 10%^4^.

**Figure 1:**
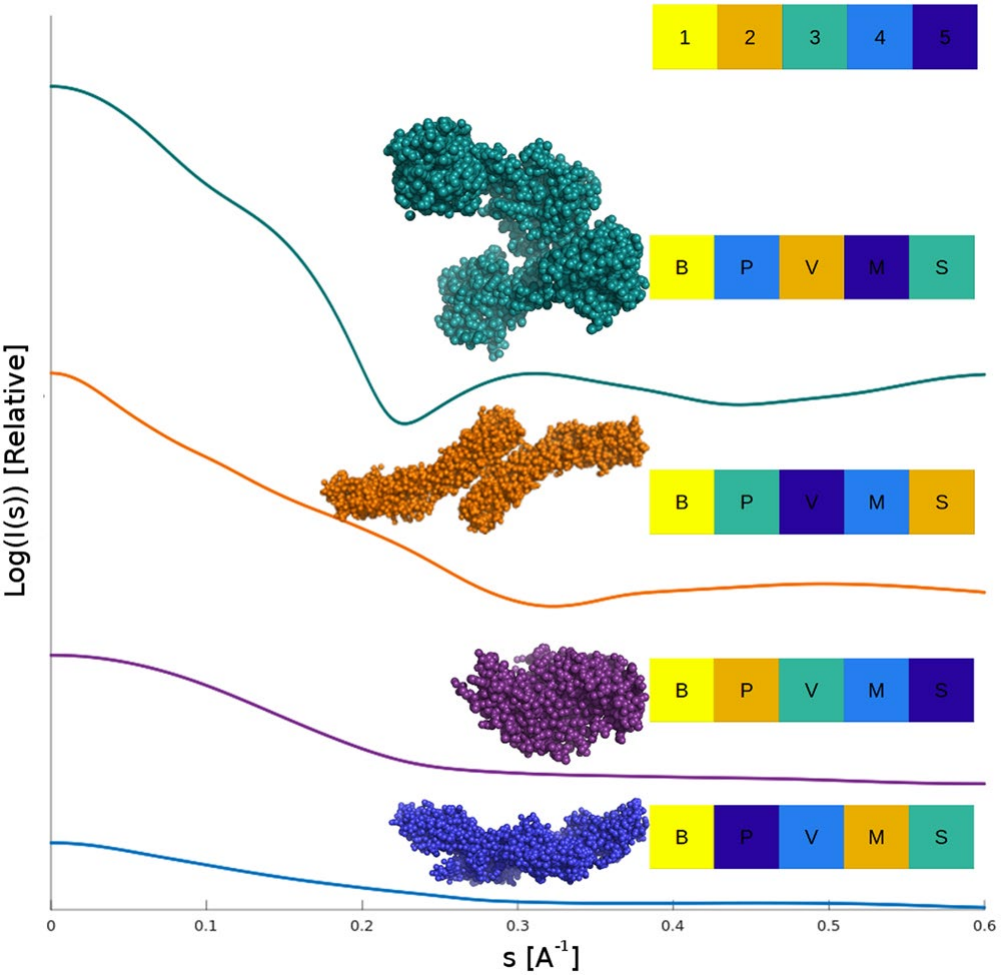
MM determination methods perform differently on different proteins. Four CRYSOL simulated SAXS profiles (Log of relative intensity against s) of proteins with different shape, the profiles are offset for clarity. These cases illustrates the variation in MM estimates of the various methods. Here each of MM_Qp_ (P), V_c_ (V), MoW (M) and Size&Shape (S) at least once provide a MM estimate with the smallest (yellow) and the largest (dark blue) relative error, respectively. However, the estimate provided by the Bayesian inference is consistently the best.

Concentration independent methods on the other hand are self-contained in that they determine the MM from a single background-subtracted scattering pattern, requiring only the *I*(0) and radius of gyration *R*_*g*_ without the need for additional measurements of the concentration or standard samples. Generally, these methods utilize the fact that the scattering profile provides information about the geometrical parameters of the solute, namely the size and volume. The available methods include the estimation of protein volume from the Porod invariant^12^, Q_p_, as implemented in the SAXSMoW tool, MoW^13^ and the empirical Volume of Correlation, V_c_^14^ originally implemented in the program ScÅtter (http://www.bioisis.net/tutorial/9/). The apparent volume obtained from Q_p_, can also be used to give an estimate of the MM, MM_Qp_, and is described here (see below). The MM_Qp_ method is not to be confused with the ‘rule-of-thumb’ MM estimates obtained from DATPOROD as implemented in ATSAS program suite^15^ which applies additional corrections to approximate the Porod volume, *V*_p_ (wherein MM ~ *V*_*p*_/1.6)^16^. More recently, a classification-based approach, Size&Shape^17^ was also proposed. For proteins, a MM can also be determined from *ab-initio* reconstructed bead models^18,19^. Each of these aforementioned methods employ different assumptions about the particle structure and utilize variable angular ranges to estimate the MM. As a result, their implementations may yield varying performance on different types of particles depending on their size, shape and experimental conditions. While each method may be applied to any data set, inherent differences might make one of them more applicable to certain cases (Fig. 1).

The concentration independent approaches require only a simple set of structural parameters obtained from the SAXS data, making them convenient and practical tools. However, there is no single, reliable and universally applicable estimator. Here, we shall first assess the performance of MoW, Size&Shape, V_c_ and MM_Qp_, excluding the *ab-initio* model approach, with calculated, noise-free, scattering patterns as well as on data with simulated experimental uncertainties. In addition, the effects of particle shape and misaligned background subtraction are evaluated to highlight the relative strengths and weaknesses of this class of methods. Building on the results of the comparison, we introduce a method which combines these diverse concentration independent MM estimators into a probability-based Bayesian^20^ estimate that consistently outperforms the individual approaches, regardless of data uncertainty, background mismatch or particle shape. The Bayesian MM estimate is accompanied with a probability score and a credibility interval that emphasizes the precision of this estimate.

## Materials and Methods

### MM determination methods

Four concentration-independent MM determination methods were considered in this study. All methods implicitly assume that the proteins are unmodified, i.e. without accounting for bound cofactors, metals or other post translational modifications.

#### MM_Qp_

The Porod invariant^12^, Q_p_, is an important characteristic of the scattering intensity and is defined as

1 $${Q}_{p}={\int }_{s=0}^{\infty }{s}^{2}I(s)ds$$

where *I*(s) is the intensity at the momentum transfer *s* = 4*π* sin (*θ*)/*λ*, with λ being the wavelength of the X-ray and 2θ the scattering angle. Assuming that the particle has a uniform scattering length density its excluded volume, V_p_ can be obtained via

2 $${V}_{p}=\frac{2{\pi }^{2}I(0)}{{Q}_{p}}$$

However, the intensities at both limits of the integral in eq. (1) have to be determined via extrapolation. During data analysis, the *I*(0) can be determined using the Guinier approximation^21^. The higher limit is evaluated up to *sR*_*g*_ = 8 with an additional extrapolation to infinity^22^. Finally, the mass estimate is obtained by dividing the volume by 1.37. This method, that we call MM_Qp_ is not the same as that implemented in *DATPOROD* of the ATSAS suite^16^, which applies additional correction factors and yields otherwise worse MM estimates (see Supplementary Material S1).

#### SAXSMoW volume correction

The approach by Fischer *et al*.^13^ also uses the scattering invariant in eq. (1) but with a different integration range (see below), and similarly determines the protein volume using the relation stated in eq. (2). Using the *I*(*s*)/*I*(0) normalised intensities, eq. (1) is integrated in a fixed range up to pre-defined values of *s*_*max*_ and empirical correction factors applied to convert the apparent volume V′ at different *s*_*max*_ into V. These correction factors were obtained from simulated SAXS profiles calculated from 1145 proteins from the PDB. The MM is determined by multiplying V by the average mass density of a typical unmodified protein (0.83 × 10^3^ kDa Å^−3^).

#### Volume of correlation

Rambo and Tainer^14^ defined the volume of correlation, V_c_ in Å^2^, based on the integrated intensity of a different scattering invariant (eq. (3)) that relates to the correlation length l_c_ eq. (4)

3 $${V}_{c}=\frac{I(0)}{{\int }_{s=0}^{\infty }sI(s)ds}$$

4 $${V}_{c}=\frac{{V}_{p}}{2\pi {l}_{c}}$$

The authors made calculations of 9446 simulated SAXS profiles from structures in the PDB where the integral was calculated up to a maximum value of *s*_*max*_ = 0.5 Å^−1^. The authors observed that the ratio V_c_^2^/R_g_ exhibits proportionality to the MM on a log-log plot, and an empirical relation was derived to relate the MM to the V_c_.

#### Size&Shape

Contrary to the other methods, Size&Shape^17^ utilizes information about size and shape of a large number of known atomic structures to infer the MM of an unknown sample, based on size and shape information derived from experimental data. Here, a shape estimate is obtained by integration of the experimental data to an apparent Volume^13^ on a normalized Kratky scale^23^ up to *sR*_*g*_ = 3, *sR*_*g*_ = 4 and *sR*_*g*_ = 5, respectively:

5 $$V^{\prime} =\frac{2{\pi }^{2}}{Q^{\prime} }\,{\rm{where}}\,Q^{\prime} ={\int }_{0}^{s{R}_{g}}{(s{R}_{g})}^{2}I(s{R}_{g})ds{R}_{g}$$

Here, similar V′ triplets for datasets indicate similar shape, but, due to normalization, they are independent of the actual size. To account for the size, the experimental *R*_*g*_ is included as additional information. To determine the MM, a weighted average of the five nearest neighbours to the point in four-dimensional size-and-shape space is given. This space is populated by 165,982 unique protein structures sourced from the PDB.

### Bayesian calculation

Bayesian inference is a way to infer the probabilities of potential values of an unknown quantity (hypotheses, H), by combining known pieces of information (evidence, E), by application of Bayes theorem. Here, we consider the MM of the protein to be the unknown quantity, and the MM estimates of the four concentration independent methods, MoW, V_c_, Size&Shape and MM_Qp_ as source of information or evidence. We infer the probabilities of potential values of the MM (… H = 10 kDa, H = 11 kDa, … H = 99 kDa, H = 100 kDa, …) using Bayes theorem:

6 $$P(H=?kDa|{E}_{M{M}_{Qp}}{E}_{{V}_{c}}{E}_{MoW}{E}_{Size\& Shape})=\frac{P({E}_{M{M}_{Qp}}|H)P({E}_{{V}_{c}}|H)P({E}_{MoW}|H)P({E}_{Size\& Shape}|H)P(H)}{P(E)}$$

**Figure 2:**
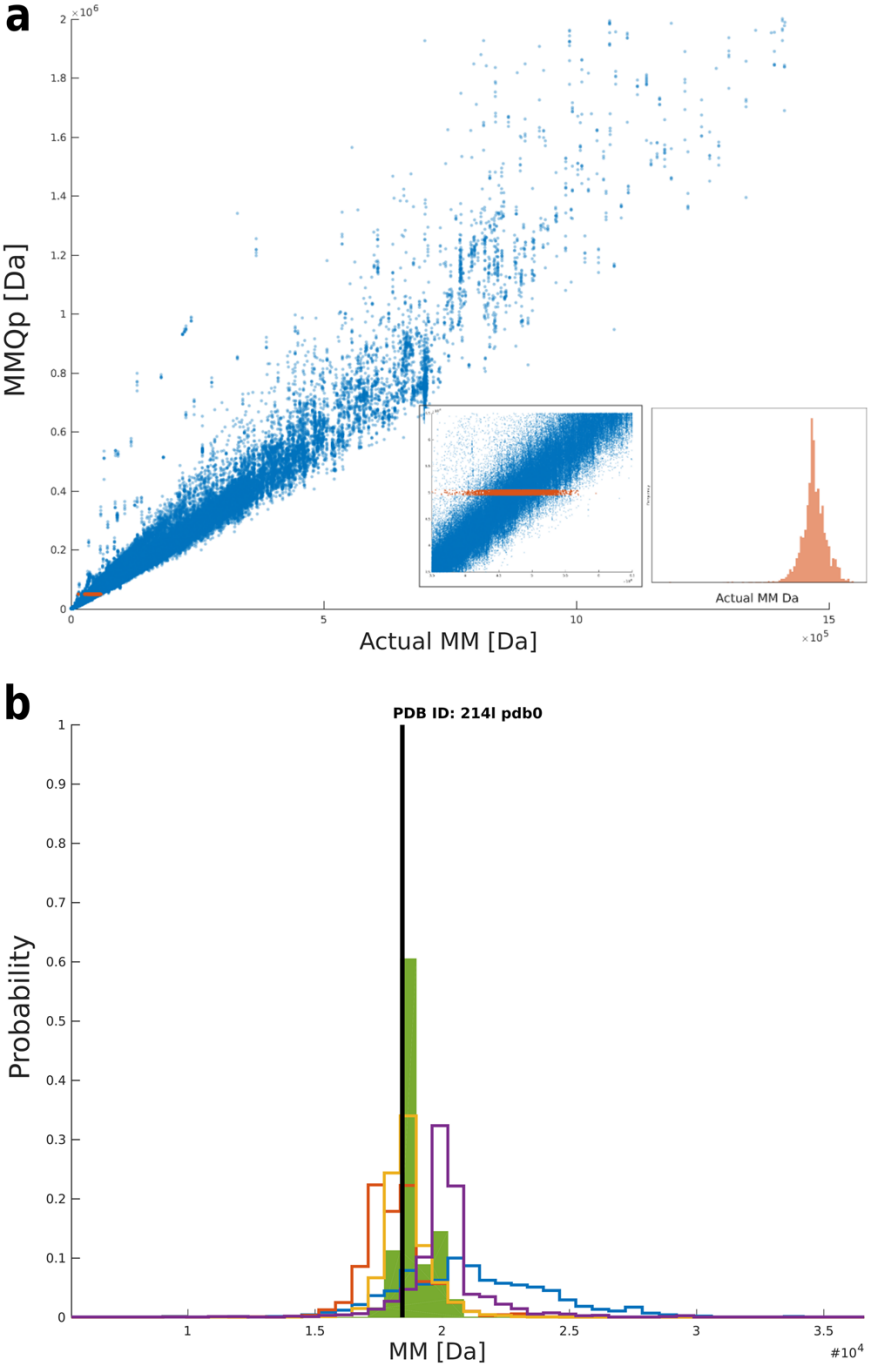
Overview of the method of Bayesian inference. (**a**) Scatter plot of actual MM (from CRYSOL) vs. the estimated MM (in this case, MM_Qp_). Given the evidence of a MM from MM_Qp_ equal to 50 kDa, a distribution is created by extracting the actual MMs (from CRYSOL) of when MM_Qp_ = 50 kDa, shown as the red points, and the corresponding distribution in the inlet figure. (**b**) Example of the Bayesian inference method for a randomly chosen protein, here PDB ID: 214l. The probability distributions of the molecular weights for each of the methods (MM_Qp_: blue; V_c_: red; MoW: yellow; Size&Shape: purple) are combined through the Bayesian calculation (green distribution). The most probable MM coincides with the actual MM (black line).

Therefore, the probability that the MM of the protein might be a certain value (for instance P(H = 10 kDa)), given the evidence of the estimates, is obtained by multiplying the respective probabilities of the observed evidence E_MMQp_, …, E_Size&Shape_ given the hypothesis and a predefined starting probability for the hypothesis, the prior P(H). Here we take all possible MMs to be equally likely, and therefore the prior P(H) has a uniform distribution. The P(E) in eq. (6) is a normalizing term, the sum of the evidence distributions. This leaves the probabilities of the observed evidence P(E|H), in other words, converting a single MM to a probability distribution. This procedure is conceptualized in Fig. 2a. Here, the actual MMs (as calculated by the program CRYSOL)^24^ that correspond to an estimate of MM_MMQp_ = 50 kDa are coloured in red. These values are subsequently binned (see below) to produce a probability distribution (inset Fig. 2a). This procedure is repeated for all methods, yielding a total of four so called likelihood distributions (P(E_MMQp_|H) * P(E_Vc_|H) * P(E_MoW_|H) * P(E_Size&Shape_|H)).

Of note, the outlined calculations have to be repeated for all hypotheses H individually. A plot of P(H|E) vs. H then yields the most likely MM, i.e. the Bayesian MM estimate, and a credible MM range i.e. the interval where the real value may, most likely, be found (Fig. 2b).

### PDB query

To obtain a sufficiently large dataset required for testing and training, a total of 223,045 atomic coordinate files describing protein structures from protein-only biological assemblies were obtained from the protein databank (PDB). Coordinate files from NMR, EM and X-ray crystallography were used. In instances where the coordinates of metals, waters, small organic or inorganic molecules and other non-protein post-translational modifications (e.g., glycosylation) occurred, these were removed from the files to produce a cohort of protein models containing only amino acids. Asymmetric units as well as biological assemblies with only a single model were selected, if they contained at least 50 amino acids. In instances where alternate conformations of amino acid side chains were included in the PDB files, only the conformation listed first in the coordinate file was used in the calculation of the SAXS profile. Finally, duplicates were removed reducing the initial pool of 223,045 protein PDB files down to 165,982 unique protein structures.

### Calculation of expected MM

CRYSOL^24^ was used to calculate the scattering profiles from the atomic coordinates of each protein structure in the training and test sets up to *s* = 0.6 Å^−1^, using 1001 data points and 30 spherical harmonics. In addition, CRYSOL reports the atomic MM from the atoms listed in the input atomic coordinate files that includes the MM contributions from hydrogens that, although not routinely reported in PDB files, are added to the atomic groups by CRYSOL to obtain the MM of the high-resolution structure.

The estimated MMs from MM_Qp_, MoW, V_c_ and Size&Shape were calculated using the corresponding DATTOOLs from the ATSAS package software suite^15^ that implement and report the methods of Fischer *et al*., Rambo and Tainer and Franke *et al.*^13,14,17^. For V_c_ and MoW, the estimate given at *s* = 0.3 Å was used. Taking the estimation of V_c_ at *s* = 0.3 Å introduces a 0.6% error as opposed to using the estimate at *s* = 0.5 Å^14^. MM_Qp_ was calculated with a DATMW available in ATSAS 2.8.3. The *R*_*g*_ and *I*(0) were determined using DATRG^16^ for noise-free data and AUTORG^16^ for scattering patterns with simulated noise (see below). Absent values, or NaNs, can result if the R_g_ and I(0) are incorrect that causes the MM calculation to fail.

### Binning

**Figure 3:**
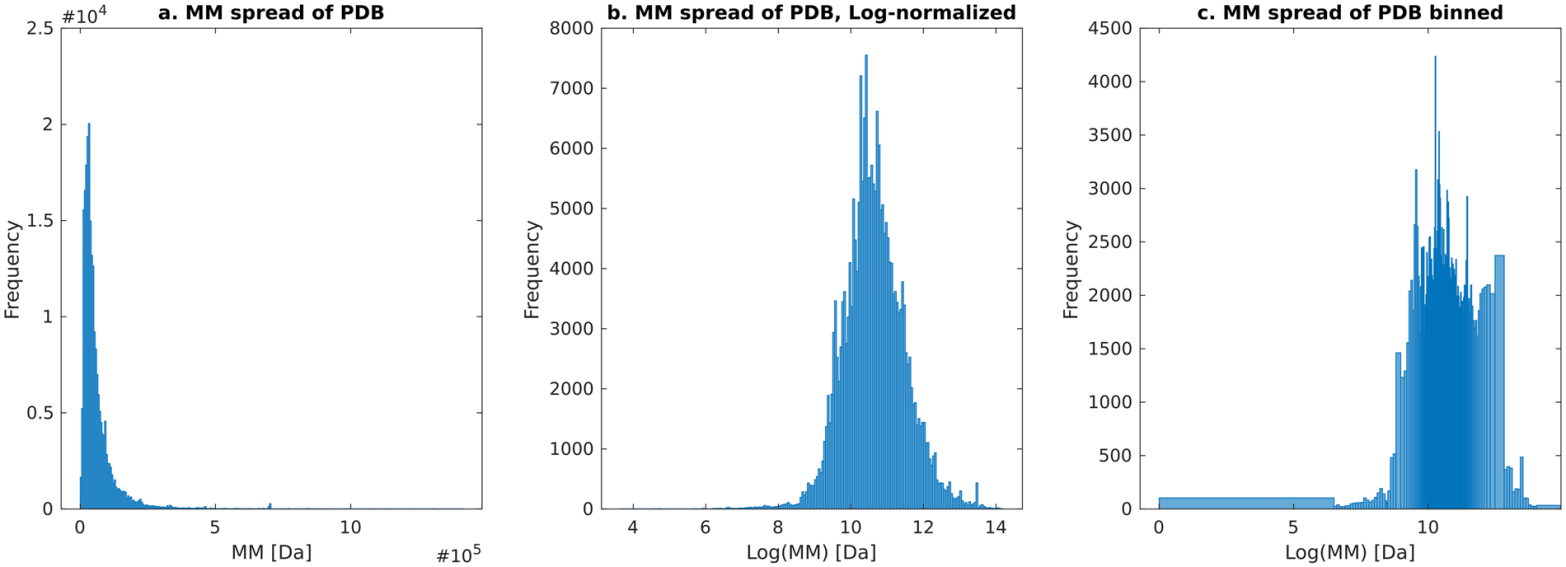
Binning procedure. (**a**) The distribution of molecular weights of the whole PDB, with very few small and large proteins. (**b**) The same dataset as to the left, but now log-normalized, with a peak at a MM of 40 kDa. (**c**) A visualization of the bins used in this study, populated with ~220,000 PDB entries. The bin-widths follow the distribution of atomic weights in the PDB (i.e. it follows the distribution in b), i.e. they vary normally on a log-scale. In the middle (around 40 kDA) the bin sizes are very small. The upper-end and lower-end tails of the distribution (corresponding to the very large/small proteins) are linearly binned to achieve a better resolution. MM’s less than 700 Da and larger than 1.30 MDa are binned to the first and last bin respectively.

To adequately describe and utilize the probability distributions in numerical calculations a binning procedure has been applied. Indeed, the binning procedure is a requirement for the application of a Bayesian model in order to represent the MMs as distributions (see previous section on Bayesian calculation). In principle, binning implies loss of information. However in the context of MM from SAXS, individual Dalton differences are not meaningful as such precision is simply not experimentally accessible. As such, it is possible to adapt a straightforward discrete binning procedure in favour of a continuous representation without compromising the usability of the method. We adapted a binning procedure reflecting the distribution of MMs of the PDB, with very small bin widths around the MM peak (40kDA) and wider bins for very large and smaller proteins (Fig. 3). To limit the loss of precision at the tails, linear bins were applied in the 5% extremes. Prior to evaluating the Bayesian inference, all bins receive a Laplace pseudo-count of one^25^ to counteract the cases where a zero-probability bin would greatly affect the outcome. The discrete binning as described here emphasizes a uniform structure count over all bins (Fig. 3).

These bins are applied in many steps throughout the Bayesian calculation. Firstly, they are used for creating the MM distributions from each of the individual methods, as seen in the inset red distribution of Fig. 2a. The bins are also used as hypothesis in the Bayesian calculation to determine the most likely MM; here, the estimated MM is taken as the centre of the bin, and the bin-width as the inherent uncertainty. Finally, when comparing the performance across the methods, the MM estimates from the test-data set (see below) are also binned, and the relative error then becomes the difference between the bin values.

### Simulation of experimental conditions

To simulate experimental noise, the approach described by Franke *et al*.^26^ was adopted. In essence, the error column of an experimental dataset was used as the source for random variations. Pseudo-random numbers were transformed to approximate Gaussian distribution with the same point-wise variations, the output of which was applied as approximations of counting errors on the simulated data producing the characteristic increase in noise at higher angles. For each simulated SAXS profile, five levels of simulated noise were applied and the MM estimates were re-calculated, yielding a total of 6 datasets. The signal to noise ratios (SNRs) were obtained by taking the median of the intensity column divided by the error column resulting in the following five SNRs: 32, 11, 4, 2 and 1. To note, this definition of SNR was adapted to quantify the difference between the datasets and should not be taken as rigid definition of SNR in general for SAXS data.

### Simulation of mismatching buffer conditions

SAXS profiles of *SNR* = 4 were used to mimic solvent mismatch, i.e., to emulate buffer over- and under-subtraction which is one of the major sources of systematic error for protein solution SAXS^1^. As incorrect buffer subtraction manifests most prominently at the higher angles, the intensity between 0.4–0.6 Å was averaged, yielding a value *I*_*average*_. Five proportions of *I*_*average*_ were then calculated and used to over or under-subtract the full SAXS profile to differing degrees by adding or subtracting, respectively, the appropriate n% value of *I*_*average*_ (where n = 0.1, 0.2, 0.4, 0.6, 0.9) at each *s*. This procedure resulted in an additional ten datasets.

### Training and test data

To determine the predictive ability of the Bayesian method while limiting overfitting, the 165,982 atomic structures were split into a training (149,084) and test (16,583) datasets for cross-validation^27^ (see Supplementary Figure S2) using MATLAB® (www.mathworks.com) function *cvpartition* that randomly selected the 16,583 cross-validation datasets from the initial pool. The training data constitute the dataset that the Bayesian method uses for extracting the underlying distributions, i.e. the probability calculations. This training data consists of 149,084 unique protein scattering curves, plus the same data at four different noise levels (the highest noise level was omitted). Of note, the Size&Shape method uses the same set of filtered 165,982 proteins for looking up the closest MM neighbours. In order not to bias the assessment of Size&Shape’s performance a modified version of DATCLASS was used, instead of using a weighted average of the first five neighbours a weighted average of the second to the sixth closest neighbours was taken. Excluding the nearest neighbour prevented the structure being queried to be used as its own neighbour.

As the performance of each MM method was tested in different contexts, such as different levels of random and systematic noise, a total of 16 test datasets were generated. These 16 datasets are comprised of the ideal data, five different levels of modeled SNRs and ten different levels of modeled solvent mismatch (see Supplementary Figure S2). For each protein in the test dataset, the four estimations of MM were used as inputs to the Bayesian estimator and the MM was calculated, all results have a sample size of 16,583.

To note, the reported performance of the Bayesian method will be slightly underestimated to the performance of the actual implementation, as the training data thereof is the union of the training and test datasets outlined here.

### Data availability

The training data generated and analysed in the current study are available from the ATSAS repository (https://www.embl-hamburg.de/biosaxs/software.html). The implementation of Bayesian method as described here, also called DatBayes, is part of the program DATMW. An explanation of training dataset used and examples of how to execute DATMW both from the command line and in PRIMUSQT^15^ is given in Supplementary Section S7.

## Results and Discussion

### Comparative study of four concentration independent MM determination methods

#### Performance on ideal simulated data

**Figure 4:**
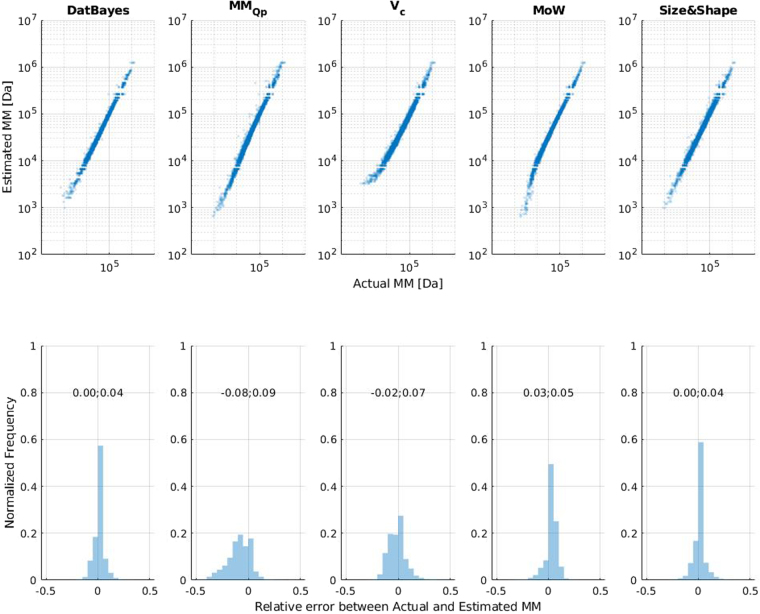
Qualitative overview of accuracy for ideal data. Dataset for ideal data with no simulated noise, dataset size is 16,563. The MM’s are expressed in terms of value of the bin (Fig. 3) which the MM falls into. *Top:* Scatter plot of the estimated MM vs Actual MM. *Bottom:* Same data as top-panel but plotted as distributions of the relative error between the actual and estimated MM. Finally the median and the median absolute deviation (mad) is shown above each distribution.

The four concentration-independent methods for evaluating the MM of proteins from solution SAXS data were compared in terms of accuracy. Fig. 4 summarizes the results by plotting actual MM against the estimated MM (top panel) as well as the normalized distribution of the magnitude of the error. All MM estimates obtained are binned as described in the methods section (Fig. 3).

In Fig. 4, the top-performing MM method, Size&Shape, can be easily identified through its narrow and peaked distribution as compared to MM_Qp_, MoW and V_c_. However, Size&Shape possesses a wide base meaning, that once the accuracy decreases, it does so rapidly. The height of the distribution of MoW is superior to that of MM_Qp_ and V_c,_ which is also reflected in the median absolute deviation (mad) value, which suggests a greater number of accurate estimates. The distributions of MoW, V_c_ and MM_Qp_ are all slightly shifted, i.e. not centered on zero (Fig. 4, bottom panel) as is indicated by their median value. In the supplementary material (Section S4), we offer correction factors for V_c_, MoW and MM_Qp_ and outline how these affect the results.

#### Performance on varying signal-to-noise data

**Figure 5:**
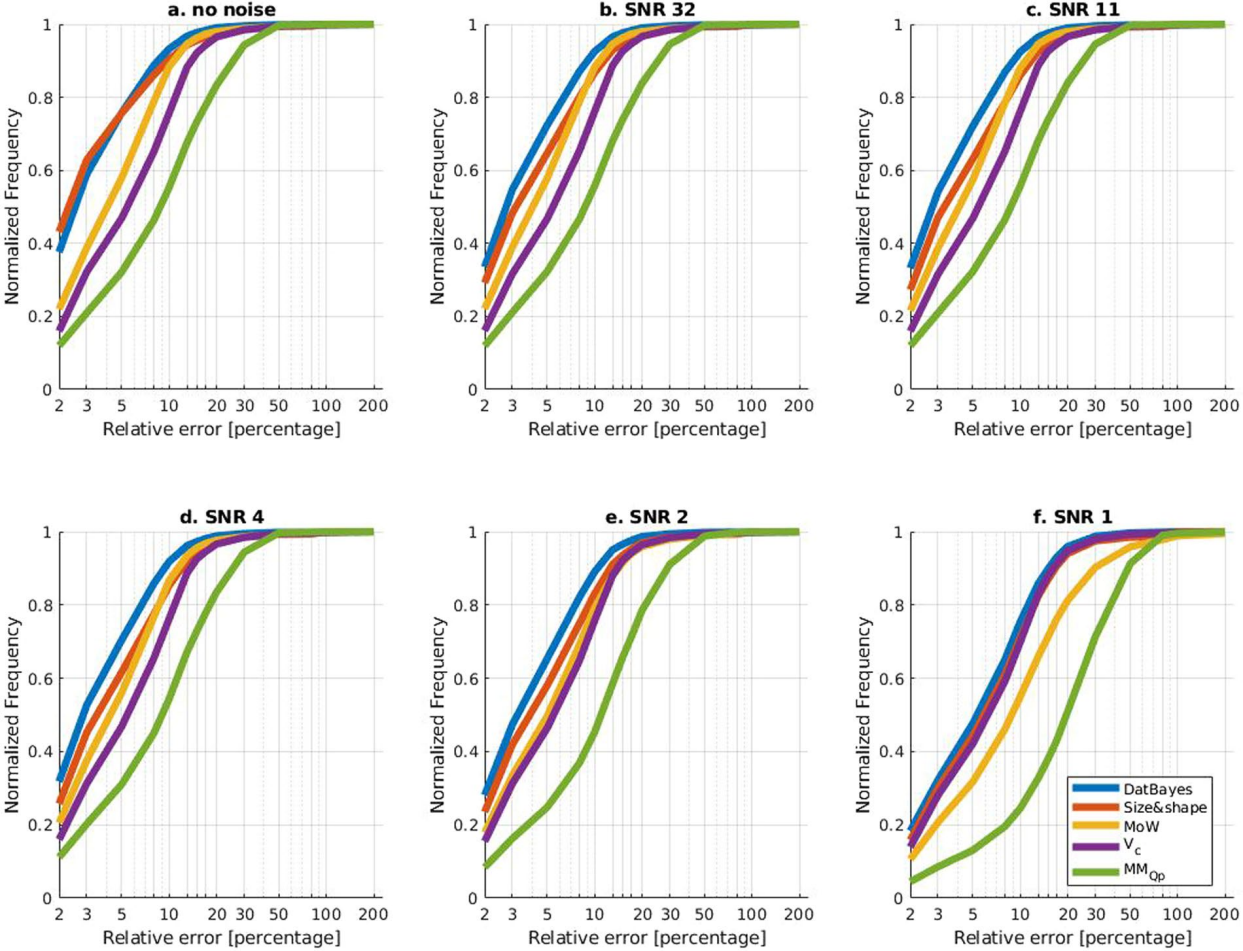
ROC-like curves for simulated random noise with different SNRs. ROC-like curves of relative error against normalized frequency. The x-axis is log-scaled to better discern the performance. (**a**) Ideal data (**b**) SNR = 32 (**c**) SNR = 11 (**d**) SNR = 4 (**e**) SNR = 2 and (**f**) SNR = 1. Methods with higher accuracy are located top-left most.

While Fig. 4 presents a qualitative overview of the performance, the more quantitative insight is given by the Receiver Operating Characteristic (ROC)-like curves^28^. Here, the magnitude of the error is plotted against the number of occurrences, meaning a very accurate method will yield a curve positioned in the upper-left corner^28^. Thus, when comparing the performance of the MM methods, the relative positioning on the ROC curve will reflect their accuracy. Fig. 5a–f shows the response of the methods to decreasing SNRs. Starting with ideal data in Fig. 5a, the ROC curve of Size&Shape assumes an upper-left position compared to the other three methods, MoW assumes a clear second place in terms of accuracy, followed by V_c_ leaving MM_Qp_ last. This ranking is generally left intact as the level of noise is increased; however the difference between MoW and Size&Shape becomes less pronounced (Fig. 5c,d). Remarkably, V_c_ remains effectively unchanged by the noise and at the lowest SNR, it is joined by Size&Shape and performs better than MoW. It is worth noting the sensitivity of Size&Shape to increased noise (Fig. 5b–d), especially the marked difference in performance when only a little noise is applied (Fig. 5b). Finally, the accuracy of MM_Qp_ worsens as the SNR is lowered, as can be seen in the right shift of the ROC-like curve (Fig. 5b–d).

#### Performance on incorrect background subtracted data

**Figure 6:**
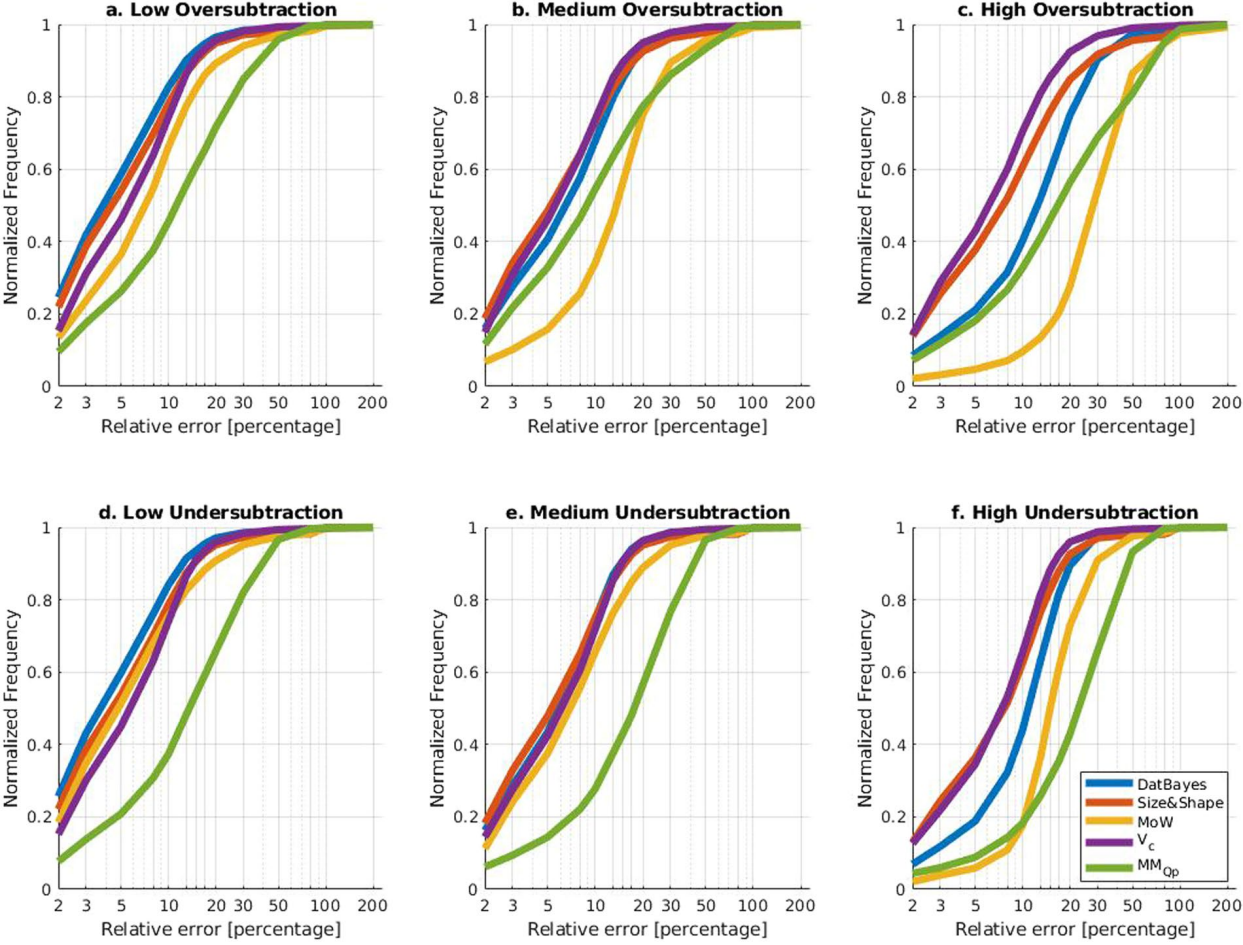
ROC-like curves of different levels of simulated systematic noise. ROC-like curves of relative error against normalized frequency for three different levels of under and over subtraction. The x-axis is log-scaled to better discern the performance Additional levels of over and under-subtraction were investigated (data not shown). Low, medium and high refers to factors of 0.1, 0.4 and 0.9 respectively (see Methods).

One of the most common sources of systematic error in experimental SAXS data is that of incorrect buffer subtraction^1^. Hence, in addition to varying SNRs, the performance of each MM method was also characterized on a total of ten datasets with five different levels of under- and over-subtraction respectively. The results are summarized in the ROC-like curves, of Fig. 6a–f (see also Supplementary Section S5). Of the MM methods, MoW is the most affected by incorrectly subtracted data, in particular over-subtracted data and to a lesser extent under-subtracted data. A similar pattern is observed for MM_Qp_, although the ROC curves indicates that MM_Qp_ is slightly more robust, as compared to MoW, against systematic errors, it is worse in terms of accuracy in most cases. The accuracy of Size&Shape is also affected by subtraction, in contrast with the previous two methods the effect is much less pronounced. However, in line with MM_Qp_ and MoW, it is also slightly more affected by over-subtraction (Fig. 6c). In contrast V_c_ is least affected by the incorrect subtraction, which is likely the result of that factor *s*, and not *s*^2^, is used in the integral evaluation (eq. (3)). This observation, taken together with V_c_’s noise resistance, suggests it a good method to consult for poor quality data.

#### Performance on different types of protein shape

**Figure 7:**
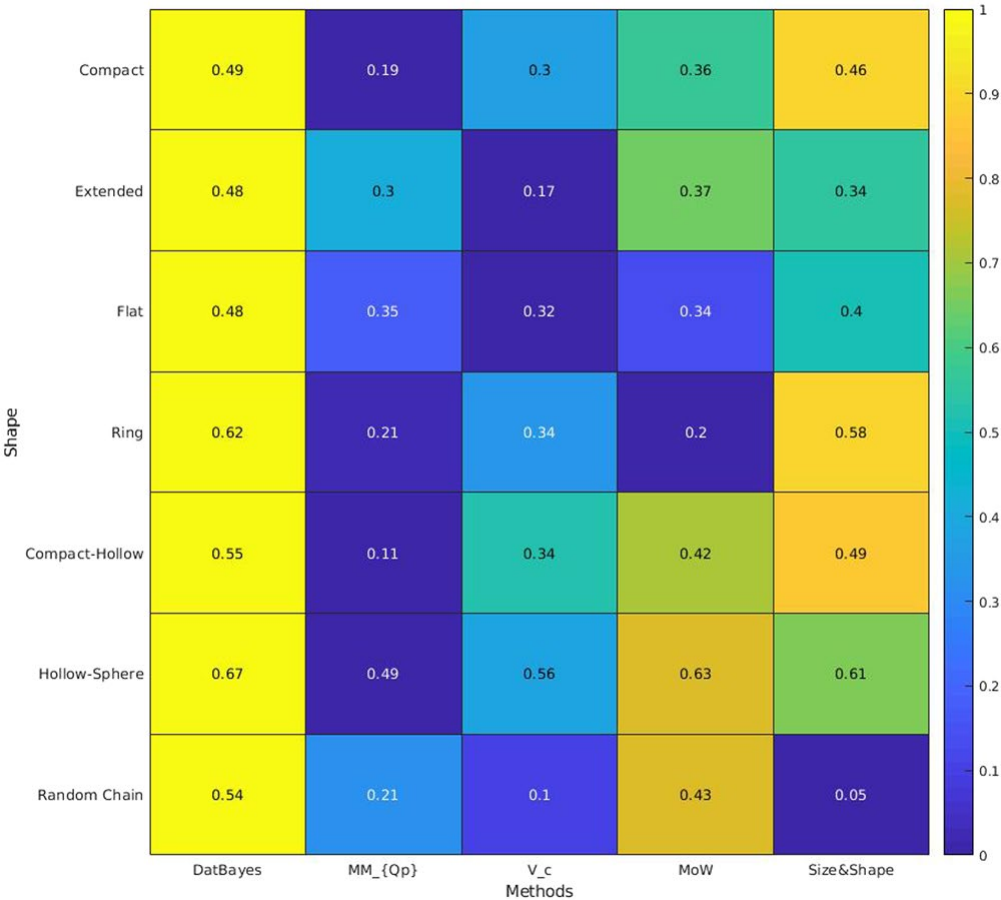
Performance of the methods for different protein shapes. Heatmap assessing the performance of the method against the protein shape, as determined by the protein classifier algorithm DATCLASS^17^. The color represents the fraction of the cases at which each method yielded the most accurate MM as determined by the smallest relative error. The figure comprises the results from all noise levels, a total of 6 noise levels each containing 16,563 unique profiles, amounting to 99,378 profiles.

Finally the performance of the four concentration-independent methods were also tested on the seven different shape classes of proteins DATCLASS can distinguish^17^: i. compact; ii. extended; iii. flat; iv. ring; v. compact-hollow; vi. hollow sphere and; vii. Random chain. The results are summarized as a heatmap in Fig. 7. The Size&Shape method ranks generally well across the shape classes, with the exception of random chains as to be expected as it refrains from giving an estimate in these cases. Interestingly, MoW seems especially apt at estimating the MM of extended proteins, while V_c_ has difficulties for these types of proteins. V_c_ ’s difficulty with extended proteins is consistent when considering that the method uses the correlation length (Methods, eq. (4)), i.e. the number average chord length of the electron density auto-correlation function^14,22^ which would be affected be extension in one dimension. All methods have difficulty with flat and ring-shaped proteins^29,30^.

#### Performance on experimental SAXS data

**Figure 8:**
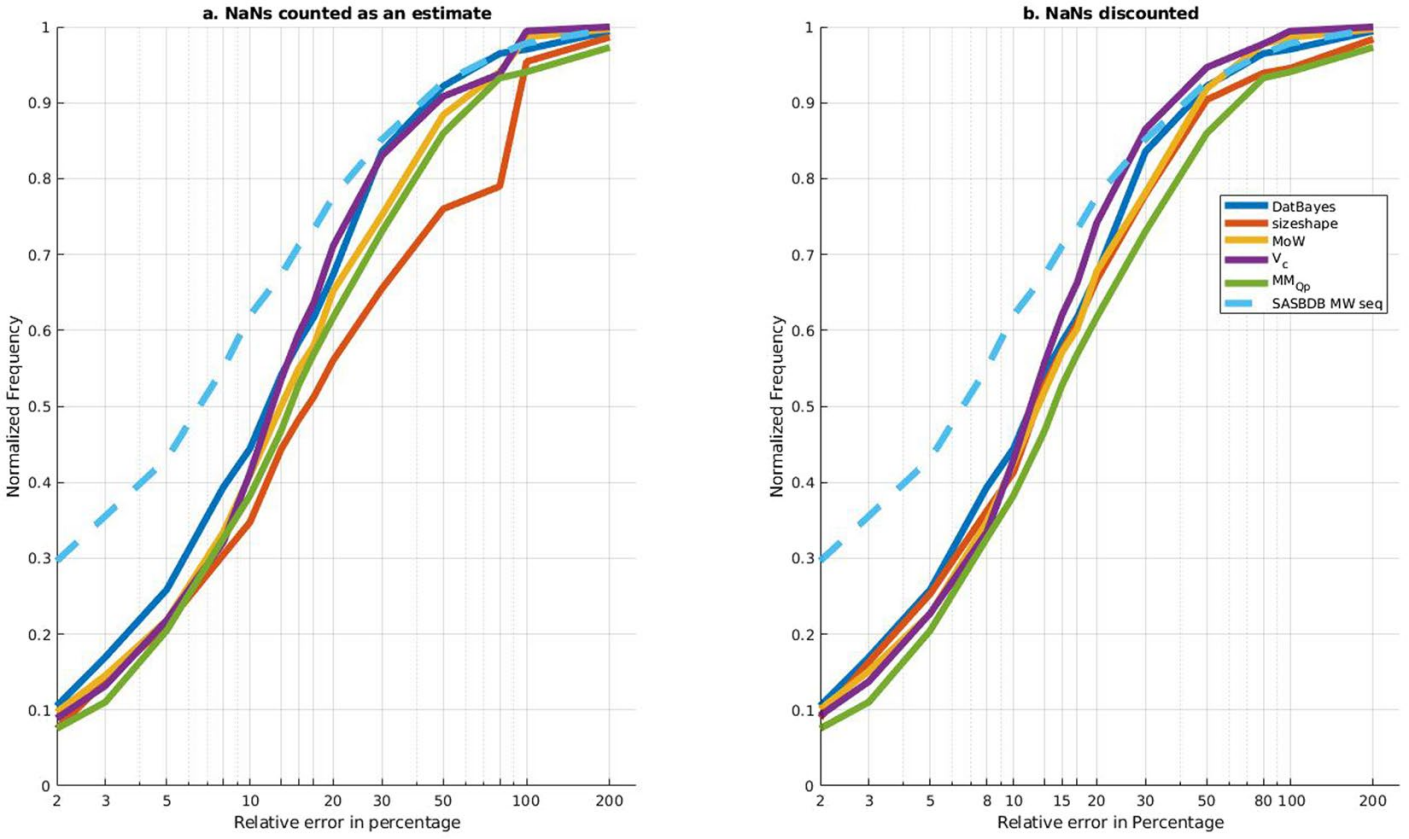
ROC-like curves for experimental data from SASBDB. ROC curves of relative error against normalized frequency for experimental data from all published SASBDB entries, 375 datasets in total. The x-axis is log-scaled to better discern the performance. The actual MM is taken to be the user submitted experimental MM. As a control, the actual MM is plotted against the MM from the user submitted sequence. *Right*: Counting NaNs as a bad estimate, and normalizing by the total number of cases. *Left:* Ignoring NaNs, normalizing by the total number of cases minus the number of NaNs.

The performance was also assessed on experimental SAXS data from SASBDB^31^ (Fig. 8), where the actual MM is taken to be the user reported experimental MM. The results from SASBDB should not be interpreted too strictly, as the control (the MM from user-submitted sequence) does not assume a position in the upper-left corner as would be expected, and therefore indicates that there is many discrepancies (Fig. 8, light-blue dashed curve). Moreover, the fraction of absent or NaN values is much larger in the case of experimental SAXS data. A NaN result is often caused by the incorrect estimation of *R*_*g*_ or *I*(0), that produce an error in the calculations performed in for instance in V_c_ or MoW which require normalization (eqs 2 and 3). Furthermore, Size&Shape does not provide MM estimates for flexible proteins due to the understandably limited set of training data available as the PDB does not represent intrinsically disordered systems. Consequently, when applied to the MM assessment of SAXS data deposited in the SASBDB, and including the NaNs, size and shape performs the worst and V_c_ the best (Fig. 8a). The performance of V_c_ could be due to the methods robustness with respect to obtaining the MM from poor quality data (see above). However, on discounting the NaNs most methods perform similarly, which perhaps MM_Qp_ being slightly worse (Fig. 8b).

### A Bayesian approach to MM determination

It is now evident that each of the four concentration-independent MM determination methods possesses their own respective strengths, however there is no consistent top-performer among them. That said, the predictive power of the individual methods may be combined using Bayesian inference, to produce a probability distribution across all possible MMs. From this distribution it is possible to determine a MM point estimate and its interval, corresponding to the highest probability and the credibility interval respectively. Here we outline the performance of the Bayesian method, or DatBayes as our implementation is called, and describe the use of credibility interval in SAXS MM analysis.

#### Accuracy of the Bayesian MM estimate

The Bayesian method is a consistent top-performer in terms of accuracy (Figs 4, 5 and 7), irrespective of SNR or shape. For ideal data only Size&Shape has a slight advantage (Fig. 5a), with a higher number of more accurate estimates, thus indicating that the remaining three methods slightly divert the Bayesian assessment. However, when considering the remaining SNRs it can be concluded that for all practical purposes, the Bayesian combination of the individual estimates is at least as good, or better, than any estimate of a single method. This statement also holds for over and under-subtracted data, with the exception at the highest degree of systematic error (Fig. 6c,f). The latter represents an extreme case, in which re-processing or even re-measurement should be considered. Furthermore, these extreme cases also produce generally lower probability scores (see Supplementary Section S6), which indirectly reflects larger credibility intervals (see below). As such, it can be said that if the sample has been prepared correctly within acceptable error, the DatBayes is the preferred method in terms of accuracy, and moreover, the probability of the MM together with the credibility interval could help to indicate, but alone cannot prove, cases with low sample/data quality. Indeed, when applied to estimating the MM from experimental SAXS data, the Bayesian approach either outperforms or is as accurate as any of the individual methods (Fig. 8).

#### The Bayesian credibility interval can be used to assess the precision of the MM estimate

**Figure 9:**
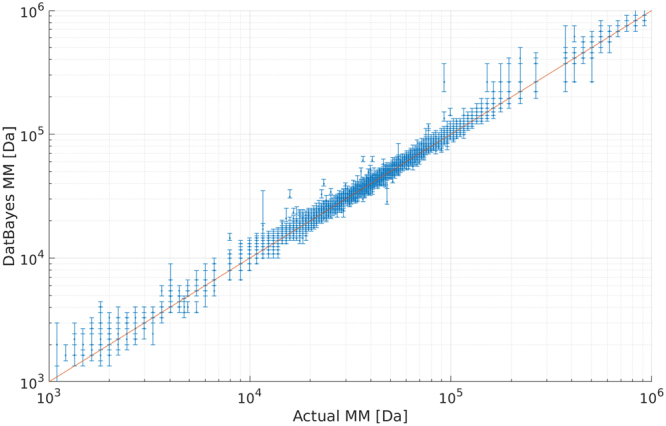
Credibility interval from Bayesian inference. Scatter plot of DatBayes MM against actual MM for ideal data. Both axis are log-scaled. The bars indicate the width of the probability distribution containing 90% of the probability mass. Note the larger bars for very small and large proteins, a result of the limited training data in these ranges of MMs.

The Bayesian method also provides a credibility interval which corresponds to the range of MMs that accumulate 90% of the probability mass. On a plot of the Bayesian MM estimate against actual MM, this credibility interval can be visualised as a bar indicating the possible MMs (Fig. 9). We observe that the actual MM is contained in the 90% empirical credibility interval (red-line plot in Fig. 9) in 86% of the test cases. In other words, in the case of a truly monodisperse solutions, the true MM of the sample is actually contained in the credibility interval 9 times out of 10. Fundamentally, the credibility interval reflects the degree of agreement on the MM of the four different methods, and its length can therefore be associated with the precision of the point MM estimate. For instance, very small and very large proteins have larger MM-ranges (Fig. 9), a result of the limited training data of such cases in the PDB and therefore an inherent error to the method. To note, a small credibility interval should not be taken as a confirmation of a good quality sample but the merely that all the methods are in consistent agreement on the MM. For instance, in the case where the DatBayes MM estimate is higher than expected, but with a narrow credibility interval, this would indicate a high potential of the sample being a mixture. However, in order to begin to delineate the presence of higher order species, or possible aggregates, a more thorough investigation^32,33^ would be necessary.

#### Prior-forwarding

The Bayesian method described so far has implemented a uniform prior, in other words assuming no prior knowledge of the sample. Alternatively, one might consider the accumulated evidence of the PDB, and use a prior following the mass distribution of the PDB (Normal distribution with a mean around 40 kDa). This prior was tested but did not yield significantly improved results (data not shown). However, in the context of a series of statistically independent, and sequentially collected data frames, as is often encountered for Size-exclusion chromatography SAXS (SEC-SAXS)^34^ or when short data frames (ms to s) are collected in succession from standard batch measurements, the uniform prior of the n-th frame may be exchanged to the posterior distribution obtained from the n-1-th frame, and used to prime the calculation of the Bayesian MM.

#### Limitations

The data used to produce the Bayesian distribution consists of simulated scattering profiles calculated from structures in the PDB. The PDB has a non-uniform distribution across shapes, i.e. a skew towards globular proteins. However, this skew has a limited effect on the performance for estimating the MM of a majority of flexible/extended proteins, such as those from SASBDB^17^, as seen by the performance of DatBayes in Fig. 8. The PDB is also used to construct the bin-sizes and their edges in the Bayesian model (Fig. 3). As the number of very small (<7 kDa) or very large (>300 kDa) structures is proportionately low, by extension the DatBayes MM estimate will be less-accurate and fall within a wide credibility interval for such samples (Fig. 9). Hence, it is always important to report both the MM credibility interval in combination with the MM estimate.

The MM_Qp_, V_c_ and MoW methods all use empirical correction factors to convert the apparent scattering volume into a mass for proteins consisting of amino acids. Although the solvation layer is, to a certain extent, taken into account by these empirical corrections, there are cases that undermine the assumption of constant volume to mass conversion, for instance where proteins are bound to metals, lipids, glycans, polynucleotides, cofactors, etc, that otherwise affect the average scattering length (and mass) density and consequently *I*(0) and the magnitude of the scattering invariants. In the case of Size&Shape, its inherent limitation in accuracy is dictated by the density of the nearest structural neighbours in the size-and-shape space. The Bayesian inference may, to a certain extent, by-pass the limitations of MM_Qp_, V_c_, MoW and Size&Shape because if any of these methods fails to produce a MM estimate then it does not contribute to the Bayesian calculation. In circumstances where a sample poses a challenge to all the individual methods, such as a heavily glycosylated flexible metalloprotein, then, by extension, such a sample will also be a challenge for DatBayes, although not in terms of deriving a MM, but rather in terms of interpreting the MM in context of the sample composition.

## Conclusions

Through a systematic analysis of over 150,000 unique SAXS patterns computed from known atomic structures, with and without simulated random noise with and without systematic experimental errors we have characterized the performance of four methods for MM determination not requiring calibration of the SAXS data. We found that these methods demonstrate variable performance depending on the size and shape of particles and the presence of random and systematic errors. Most importantly we found that these differently performing individual methods can be meaningfully combined using Bayesian inference. The Bayesian combination was demonstrated to be consistent top-performer over each individual method yielding a MM estimate within 10% of the expected value in over 90% of all cases regardless of experimental noise or particle shape. The Bayesian method also has the added benefit of providing more detailed a credibility interval reflecting the precision of the estimated MM.

Each MM determination method utilizes its own physical and mathematical assumptions, and therefore harbours its own particular advantages and limitations. The Bayesian estimate effectively combine these methods utilizing their strengths and suppressing the shortcomings of the individual techniques, and is therefore superior to any single method. The Bayesian model provides a first step, or proof of principle, that the four methods can be meaningfully combined without making assumptions about the nature of the distributions, such as their normality. Further, pertaining to the disproportionate size distribution in the PDB a possible avenue of improvement is the consideration of different binning strategies, for instance optimizing the bin-edges. It will further be straightforward to test and include other MM determination methods in the Bayesian assessment when/if these will become available. It should be noted that the Bayesian inference method as shown here was deliberately trained on a set of ~149,000 scattering patterns, leaving the other available structures for testing. The public distribution of the program will employ all the information available in the PDB, meaning the results should only improve.

The concentration independent Bayesian method is robust and can be directly compared to a concentration dependent MM estimate if the latter is available. In circumstances where the protein concentration cannot be accurately determined, such as for in-line size-exclusion chromatography SAXS^34,35^, the proposed approach offers a reliable MM assessment. The described method as outlined in this manuscript has been implemented in the program DATMW, and is currently available in the ATSAS 2.8.3 release, which is freely available for academic users (https://www.embl-hamburg.de/biosaxs/software.html).

## Electronic supplementary material

Supplementary information

## References

[1] Jeffries CM (2016). Preparing monodisperse macromolecular samples for successful biological small-angle X-ray and neutron-scattering experiments. Nat. Protoc..

[2] Kikhney AG, Svergun DI (2015). A practical guide to small angle X-ray scattering (SAXS) of flexible and intrinsically disordered proteins. FEBS Letters.

[3] Dyer KN (2014). High-throughput SAXS for the characterization of biomolecules in solution: A practical approach. Methods Mol. Biol..

[4] Mylonas E, Svergun DI (2007). Accuracy of molecular mass determination of proteins in solution by small-angle X-ray scattering. J. Appl. Cryst.

[5] Trewhella J (2017). 2017 publication guidelines for structural modelling of small-angle scattering data from biomolecules in solution: An update. Acta Crystallogr. Sect. D Struct. Biol..

[6] Grishaev, A. Sample preparation, data collection, and preliminary data analysis in biomolecular solution X-ray scattering. *Curr. Protoc. Protein Sci*. Chapter **17**, 17.14.1-17.14.18 (2012). 10.1002/0471140864.ps1714s70 PMC3523188 23151743

[7] Kratky O, Porod G, Kahovec L (1951). Einige Neuerungen in der Technik und Auswertung von Röntgen-Kleinwinkelmessungen. *Zeitschrift für Elektrochemie und Angew*. Phys. Chemie.

[8] Guinier A (1939). La Diffraction des rayons x aux très petits angles: application à l’étude des phénomènes ultramicroscopiques. Ann. Phys..

[9] Dreiss CA, Jack KS, Parker AP (2006). On the absolute calibration of bench-top small-angle X-ray scattering instruments: A comparison of different standard methods. J. Appl. Crystallogr..

[10] Orthaber D, Bergmann A, Glatter O (2000). SAXS experiments on absolute scale with Kratky systems using water as a secondary standard. J. Appl. Crystallogr..

[11] Allen AJ, Zhang F, Joseph Kline R, Guthrie WF, Ilavsky J (2017). NIST Standard Reference Material 3600: Absolute Intensity Calibration Standard for Small-Angle X-ray Scattering. J. Appl. Crystallogr..

[12] Porod G (1951). Die Röntgenkleinwinkelstreuung von dichtgepackten kolloiden Systemen - I. Teil. Kolloid-Zeitschrift.

[13] Fischer H, De Oliveira Neto M, Napolitano HB, Polikarpov I, Craievich AF (2010). Determination of the molecular weight of proteins in solution from a single small-angle X-ray scattering measurement on a relative scale. J. Appl. Crystallogr..

[14] Rambo RP, Tainer JA (2013). Accurate assessment of mass, models and resolution by small-angle scattering. Nature.

[15] Franke D (2017). ATSAS 2.8: A comprehensive data analysis suite for small-angle scattering from macromolecular solutions. J. Appl. Crystallogr..

[16] Petoukhov MV (2012). New developments in the ATSAS program package for small-angle scattering data analysis. J. Appl. Crystallogr..

[17] Franke, D., Jeffries, C. M. & Svergun, D. I. Data Mining in Structural Biology: Machine Learning Methods for Data Analysis of Biological Macromolecules in Solution. *Biophysical Journal.*10.1016/j.bpj. (2018). 10.1016/j.bpj.2018.04.018 PMC6129182 29874600

[18] Franke D, Svergun DI (2009). DAMMIF, a program for rapid ab-initio shape determination in small-angle scattering. J. Appl. Crystallogr..

[19] Svergun DI (1999). Restoring low resolution structure of biological macromolecules from solution scattering using simulated annealing. Biophys. J..

[20] Gelman, A.* et al.**Bayesian Data Analysis, Third Edition*. CRC Press (2013).

[21] Guinier A, Fournet G (1955). Small angle scattering of X-rays. J. Polym. Sci..

[22] Feigin, L. A. & Svergun, D. I. *Structure Analysis by Small-Angle X-Ray and Neutron Scattering.* Plenum Press (1987).

[23] Durand D (2010). NADPH oxidase activator p67phox behaves in solution as a multidomain protein with semi-flexible linkers. J. Struct. Biol..

[24] Svergun D, Barberato C, Koch MH (1995). CRYSOL - A program to evaluate X-ray solution scattering of biological macromolecules from atomic coordinates. J. Appl. Crystallogr..

[25] Manning, C. D., Raghavan, P. & Schutze H. *Introduction to Information Retrieval*. Cambridge University Press (2008).

[26] Franke D, Jeffries CM, Svergun DI (2015). Correlation Map, a goodness-of-fit test for one-dimensional X-ray scattering spectra. Nat. Methods.

[27] Hastie, T., Tibshirani, R. & Friedman, J. *The Elements of Statistical Learning: Data Mining, Inference, and Prediction, Second Edition**(Springer Series in Statistics)*. Springer; 2nd ed. 2009. Corr 7th printing 2013 edition (2011).

[28] Zweig, M. H. & Campbell, G. Receiver-operating characteristic (ROC) plots: a fundamental evaluation tool in clinical medicine. *Clin. Chem.* **39**, 561–577 (1993). 8472349

[29] Volkov VV, Svergun DI (2003). Uniqueness of ab initio shape determination in small-angle scattering. in. Journal of Applied Crystallography.

[30] Petoukhov MV, Svergun DI (2015). Ambiguity assessment of small-angle scattering curves from monodisperse systems. Acta Crystallogr. Sect. D Biol. Crystallogr..

[31] Valentini E, Kikhney AG, Previtali G, Jeffries CM, Svergun DI (2015). SASBDB, a repository for biological small-angle scattering data. Nucleic Acids Res..

[32] Onuk AE (2015). Constrained Maximum Likelihood Estimation of Relative Abundances of Protein Conformation in a Heterogeneous Mixture From Small Angle X-Ray Scattering Intensity Measurements. IEEE Trans. Signal Process..

[33] Konarev, P. V. *et al*. PRIMUS: a Windows PC-based system for small-angle scattering data analysis. *J. Appl. Crystallogr*. **36**, 1277–1282 (2003).

[34] David G, Pérez J (2009). Combined sampler robot and high-performance liquid chromatography: A fully automated system for biological small-angle X-ray scattering experiments at the Synchrotron SOLEIL SWING beamline. J. Appl. Crystallogr..

[35] Graewert MA (2015). Automated Pipeline for Purification, Biophysical and X-Ray Analysis of Biomacromolecular Solutions. Sci. Rep..

