# Supplementary material for: Consensus Bayesian assessment of protein molecular mass from solution X-ray scattering data
Source: Sci Rep. 2018 May 8;8:7204. doi: 10.1038/s41598-018-25355-2 (PMC5940760; doi:10.1038/s41598-018-25355-2)
Supplement: Supplementary file 1 — Supplementary information [file 41598_2018_25355_MOESM1_ESM.pdf]

Supplementary material

Consensus Bayesian assessment of protein molecular mass from solution X-ray scattering data

Nelly R. Hajizadeh, Daniel Franke, Cy M. Jeffries, Dmitri I. Svergun

S1. Distinguishing the MM obtained from the Porod volume from the "total fluctuation" method

The Porod invariant can be used to estimate the so called Porod volume of the particle. This procedure assumes that the protein is homogenous, and therefore expects the intensity I(s) to decay proportionally to s^{-4}. Only when this assumption holds will the Porod invariant will be proportional to the volume of the particle. However, particles are not necessarily homogeneous, and to compensate for this, the implementation of Porod in ATSAS 2.8.2 (and earlier versions) subtract a constant to force the I(s) to decay proportional to s^{-4}. The volume obtained from this procedure is subsequently divided by a factor of 1.6 to arrive at the MM. In this investigation, a method which uses the Porod invariant, but not the porod approximation, MM_{Qp}, was used to determine the MM. The MM_{Qp} method performs superior to that of the MM estimated obtained using the Porod approximation, Porod MM, as seen in Figure S1 which plots the estimated MM against the actual MM. The spread of MM_{Qp} method is significantly smaller than that of Porod.

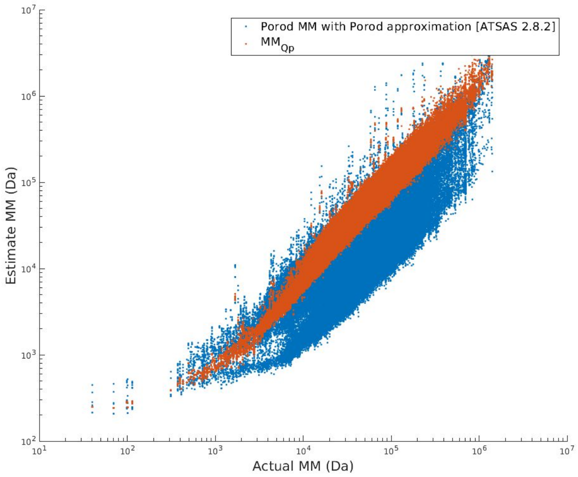

Figure S1 - *Comparison of MM_{Qp} versus Porod.* Scatter plot of estimated MM against actual MM for 99 898 proteins at six different SNRs. Blue dataset corresponds to the estimate from Porod as calculated with the DatPorod program in ATSAS, 2.8.2 (which applies Porod approximation). MM_{Qp} uses the Porod scattering invariant, but does not apply the Porod approximation to the volume calculation.

S2. Overview of datasets with simulated data

The PDB was mined resulting in ~220,000 PDB files, and after removing duplicates ~165,000 files remained. Of these ~165,000, a test data-set containing ~16k of scattering curves were produced. The remaining ~149 were used as training for the Bayesian. To determine the performance on different types of random and systematic noise, these deviations were added to the test-dataset. Yielding a total of 5 more datasets with different levels of random noise, and 10 more datasets with 5 different levels of under and over subtraction respectively.

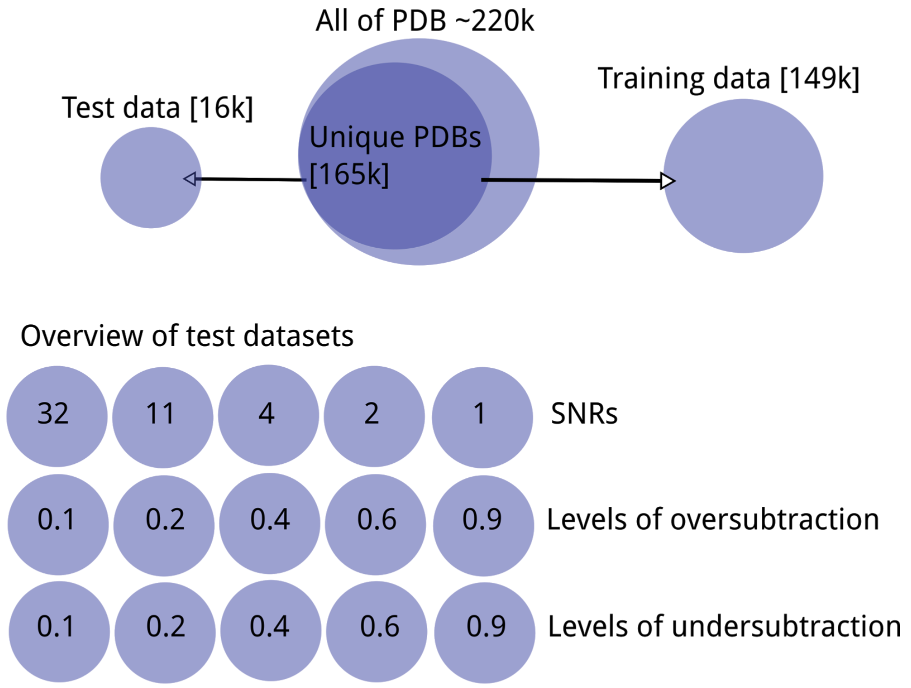

Figure S2 - *Overview of datasets used in this study.* Initial set of PDB structures were filtered down to unique structures, and divided up into training and test data for cross-validation. The training data consists of ideal data, and data from four different noise levels (the highest excluded). The test data contains ideal data, plus five and ten datasets for random and systematic noise respectively.

S3. Binning the data: Another view on estimate vs. actual MMs

The distribution of actual MM against the estimated MM, in this case of MoW. A good method has a narrow strip, with strong linear relationship. When the incoming estimate of MoW is, for instance 50 kDA, this value is binned, obtaining a bin number of for instance 60. The strip vertical strip corresponding to an estimated MM of bin equal to 60 is then taken, which yields all corresponding actual MMs. From these actual MMs, a probability distribution is built. Hence, the wider the strip, the wider the resulting distributions become. A wider distribution has less confidence in the estimated MM, as the probability is ‘smeared’ across many possible MM as opposed to being rather defined.

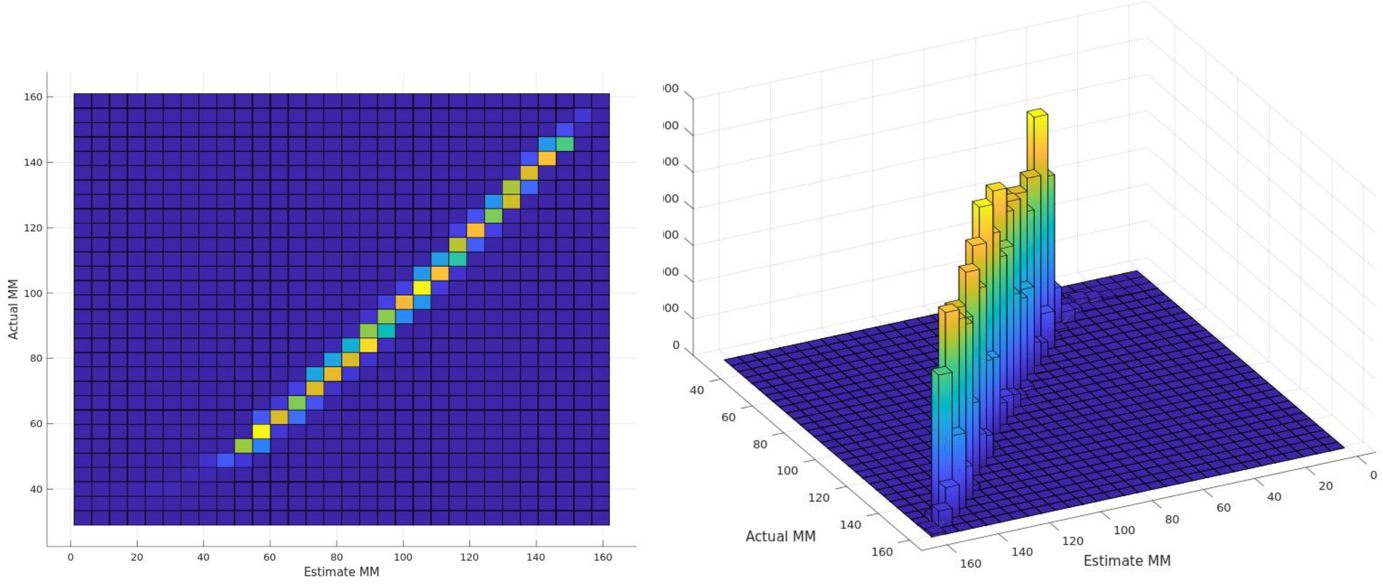

Figure S3 - *Distribution MoW MM estimates against actual MM.* Three dimensional distribution of the actual MM against the estimated MM from MoW. Left: Top view, Right: Side view, showcasing the height of the distributions. The axis are in units of bin number (and not value, which has units of Dalton).

S4. Correction factors for MoW, V_c and MM_{Qp}

Using all training data (which is composed of ideal data, in addition to the same data at four different noise levels) with an atomic MM between 8kDa and 1.2MDa, to ensure that extreme outliers do not disproportionately affect the correction, the mean of the atomic MM to the estimate MM was taken, resulting in the following factors for MM_{Qp}, V_c and MoW: 1.1020, 1.0253, 0.9761, respectively. The application of the correction factors shifts the mean of the distribution to zero (Figure S4, bottom panel: blue datasets).

The most affected is MoW which, after correction, performs with similar accuracy to that of SizeShape (Figure S4.1, panel B, C, D). In addition, correcting MoW makes it more resistant to under/over-subtraction (Figure S4.2, panel B, E).

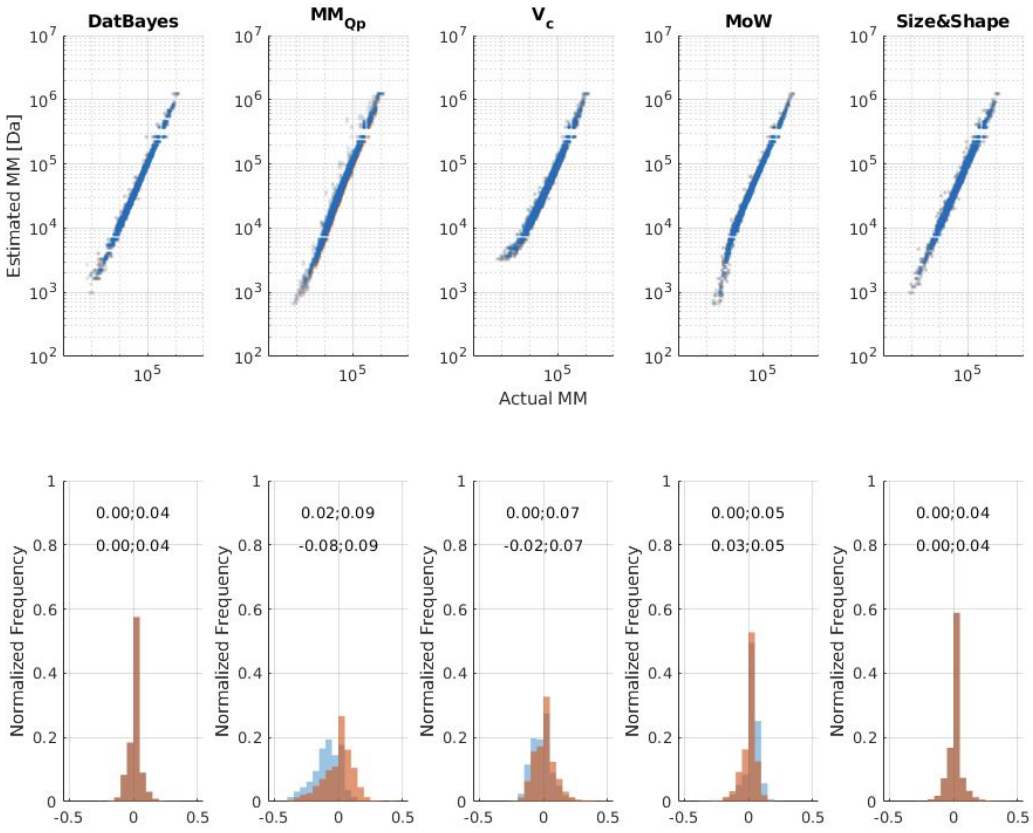

Figure S4 - *Comparing corrected and uncorrected ideal data.* Dataset with applied correction factor is shown in blue, and dataset without correction factors in red, dataset size is 16 583. The median and the mad value is shown for both the corrected (top) and uncorrected (bottom) datasets. *Top:* Scatter plot of the estimated MM vs Actual MM. *Bottom:* Same data as top-panel but plotted as distributions of the relative error. Correction factor applied for MoW, V_c and MM_{Qp} centers the distributions on or close to zero.

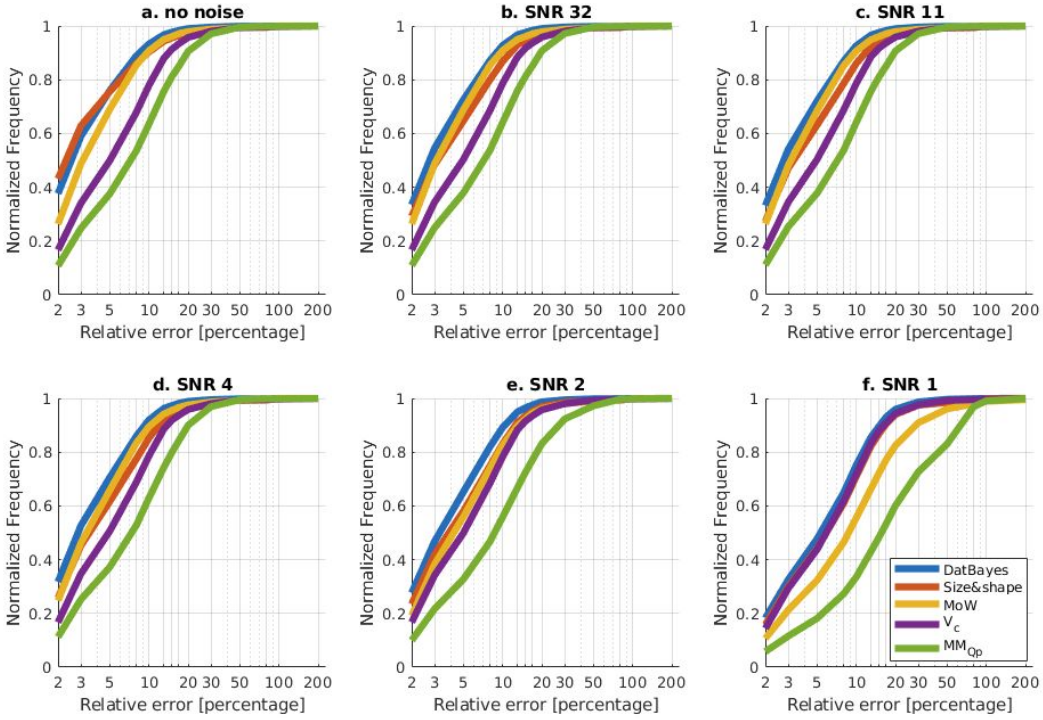

Figure S4.1 - *Response to noise with corrected methods.* ROC-like curves of number of relative error against normalized frequency. The relative error is expressed as percentage difference between the binned actual and estimated MM. The x-axis is log-scaled to better discern the performance. *a)* ideal data *b)* SNR = 32 *c)* SNR = 11 *d)* SNR = 4 *e)* SNR = 2 and *f)* SNR = 1. Methods with higher accuracy are located top-left most.

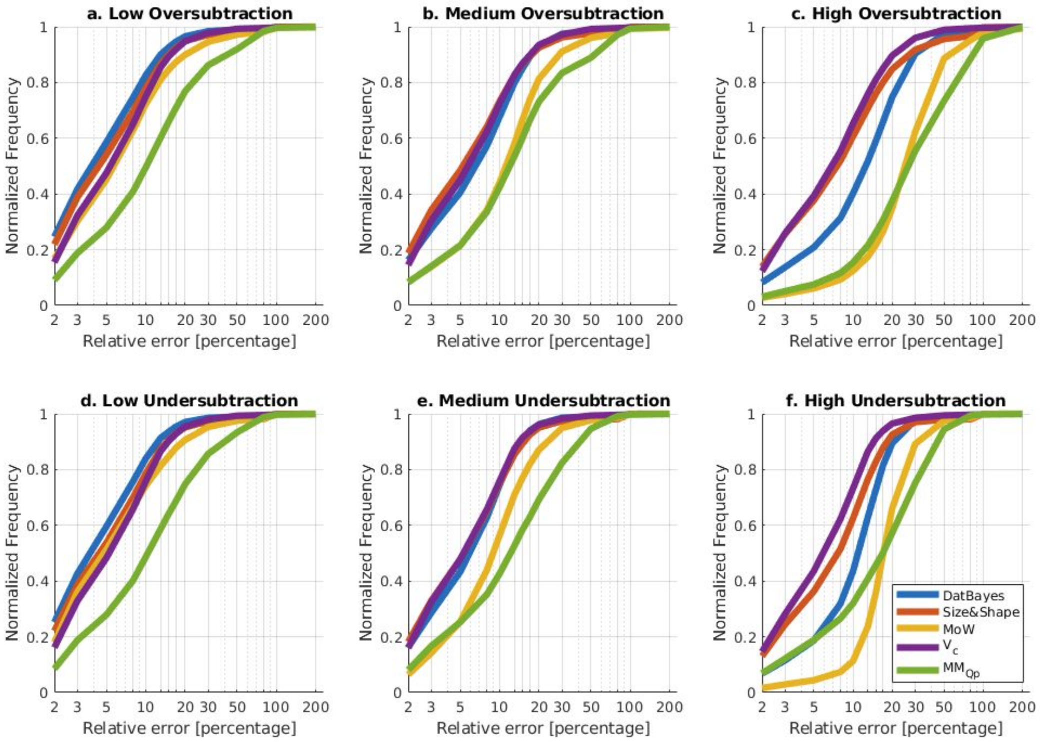

Figure S4.2 - *Response to systematic errors for corrected methods.* ROC-like curves of relative error against normalized frequency for three different levels of under and over subtraction. The relative error is expressed as percentage difference between the binned actual and estimated MM. Additional levels of over and under-subtraction was investigated (data not shown). Low, Medium and High refers to factors of 0.1, 0.4 and 0.9 respectively (see Methods).

S5. ROC-like curves for incorrect buffer subtraction

To further examine the response of the individual methods to systematic deviations, i.e. under and over-subtracted data, we complement Figure 6 in the manuscript with Figure S5, which contains the same data as in Figure 6 (i.e. uncorrected), but with panels which cluster by method rather than by degree of systematic noise.

The systematic deviations (dotted and dashed lines) are compared to the normal data (blue solid line). As mentioned in the results, MoW shows the greatest aversion to this type of systematic deviation, as can be seen by the most aggressive response. Interestingly, over-subtraction decreases the accuracy markedly in comparison to under-subtraction (dashed vs. solid lines). Some degree of over-subtraction seems to improve MM_{Qp}, reinforcing the need for a correction factor. Size&Shape is equally affected by over and undersubtraction. Finally, V_c is overall least affected by incorrect background with under-subtraction affecting its performance the most.

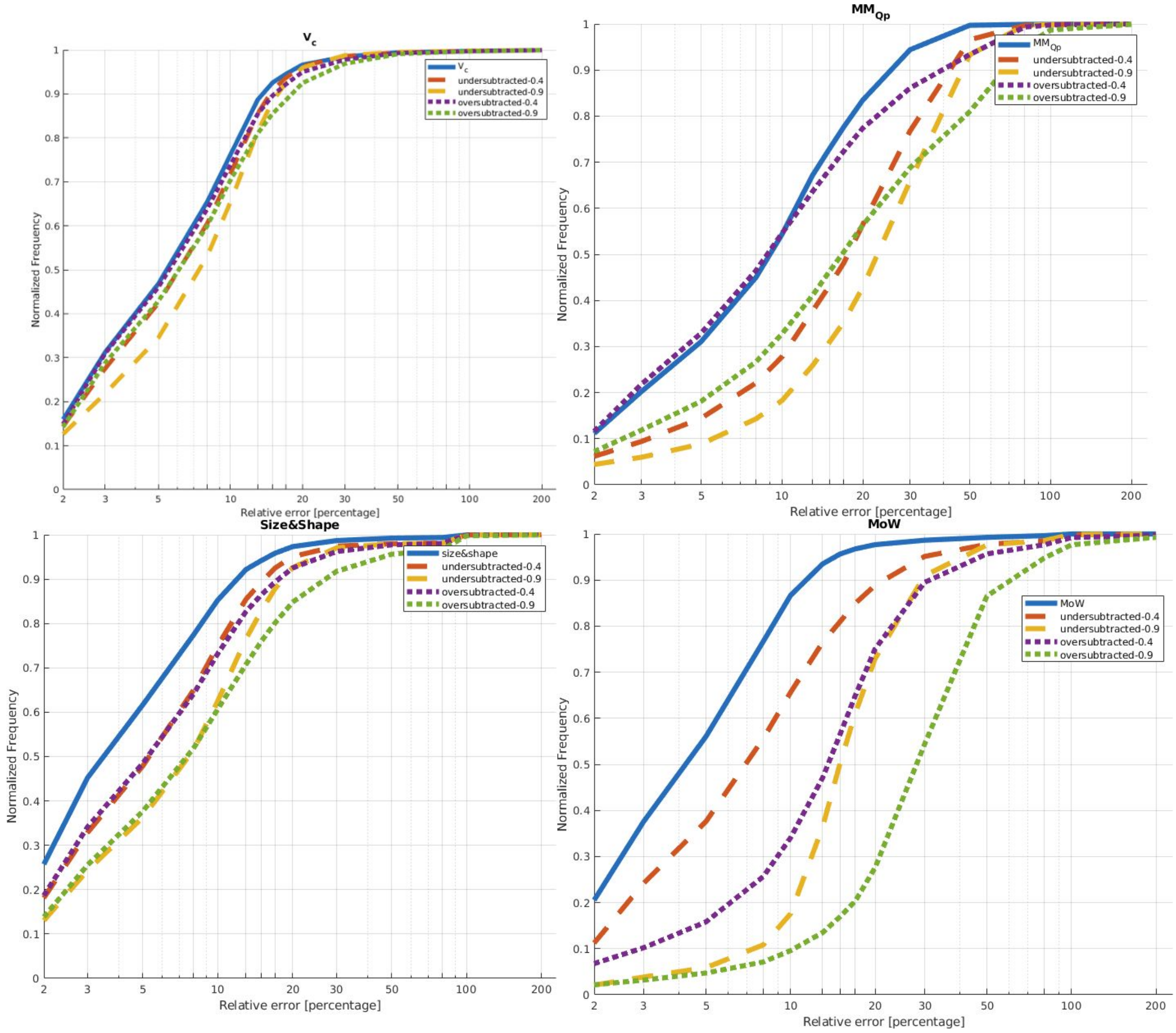

Figure S5 - *Another look at the effect of systematic noise*. ROC-like curve for the four methods: Size&Shape, $\mathrm{MM_{Qp}}$, MoW and V_c. The curves were generated using a set of under-subtracted (dashed lines), under-subtracted (dotted lines) datasets. For comparison, the data with simple random noise (SNR = 4) is shown in solid blue line.

S6. Probability distributions for highly oversubtracted data vs. ideal data

For highly over- and undersubtracted data the Bayesian method is not a top-performer in terms of accuracy. This is because the training data used to build the distributions for the Bayesian are built with ideal and noisy data, therefore when the MM estimation of the test-data has been shifted, there will be a mismatch between these datasets. The lower performance of the Bayesian method on the highest degree of systematic deviation is reflected in the probability (Figure S6) that accompanies the MM estimate. The dataset with high under-subtraction has a probability distribution that is shifted towards lower scores (Figure S6, red), with a larger spread (Figure S6, red scatter plot), not to mention many serious outliers with low probability scores. The Bayesian probability is an indication of the overlap between the four distributions and is therefore also related to the credibility interval. As such, a general shift towards lower probabilities indicate an overall lower agreement of the methods. In this case this shift is due to to a 'poor sample', however as mentioned in the main-text, a low probability score/large credibility interval cannot be taken as a proof of low data/sample quality. It may merely guide the user who wishes to investigate their sample more thoroughly before modeling. Here, the shift could be noticed, most likely due to difference in how the methods are affected by under/over subtraction. One could, however, imagine a case in which a sample contains considerable aggregates, but where all methods agree unisonly on a MM. Here, the user would obtain a MM estimate from the Bayesian method which has a high probability and possibly a small interval. We would therefore like to stress the importance of considering the Bayesian MM point estimate, its credibility interval and probability together in the context of sample composition, and with the aid of additional information such as concentration dependent MMs estimates, the quality of the guinier region etc.

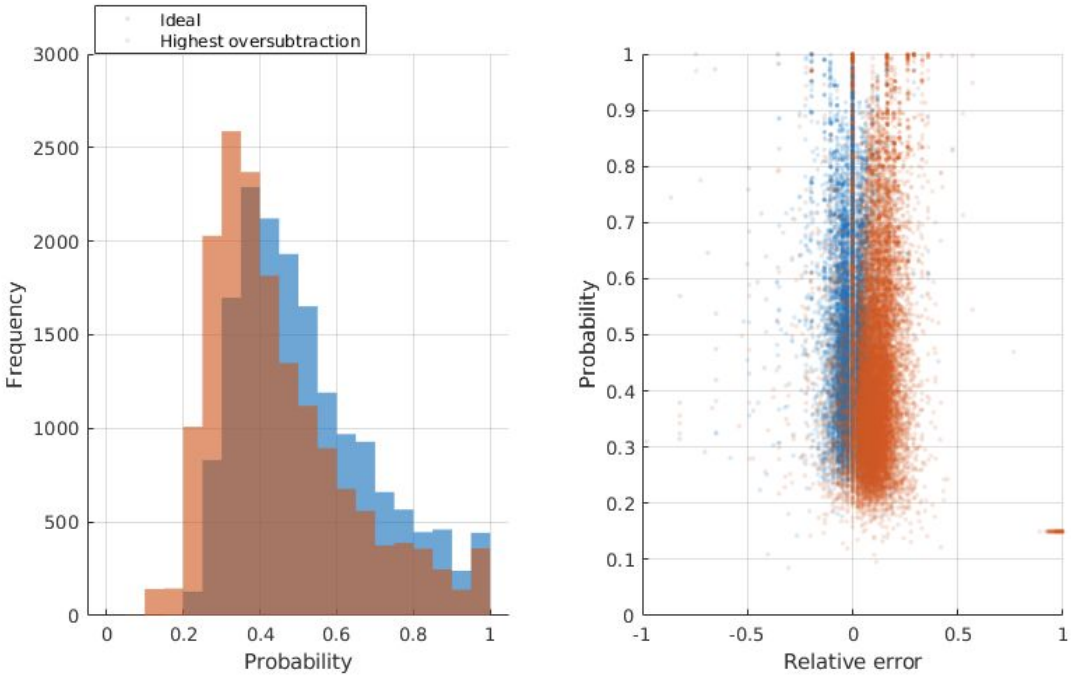

Figure S6 - *Comparing highly oversubtracted data to ideal data. Right*: Comparison of the probability distributions of ideal (blue) vs. highly over subtracted data (red). *Left:* Relative error in terms of bin difference versus probability. Note narrowing of spread at higher probabilities, and right-shift of the relative errors from the oversubtracted data.

S7. Data availability and program access

S7.1 Running DatBayes

DatBayes is part of the ATSAS program DATMW, and can be run from the command line or the PrimusQT window as described below. Both a .dat file and an .out file can be used.

Command-line

```
datmw bsa_hepes_0004.dat --i0 5.15e+03 --rg  3.01 --method=bayes

Output: MW (Da), MW Score, CI lower, CI upper, CI prob., file name

74325.0       0.450798       69650.0       77400.0       0.928273
bsa_hepes_0004.dat
```

PrimusQT window

1. Load your data-file into PRIMUS QT
2. Right-click on the file, and choose 'Molecular weight'
3. A Guinier wizard will appear, choose your I(0) and Rg and click next
4. The MM estimate from the individual methods are shown in the top, and the Bayesian estimate together with the point estimates probability, credibility interval and the probability of the credibility interval is shown.

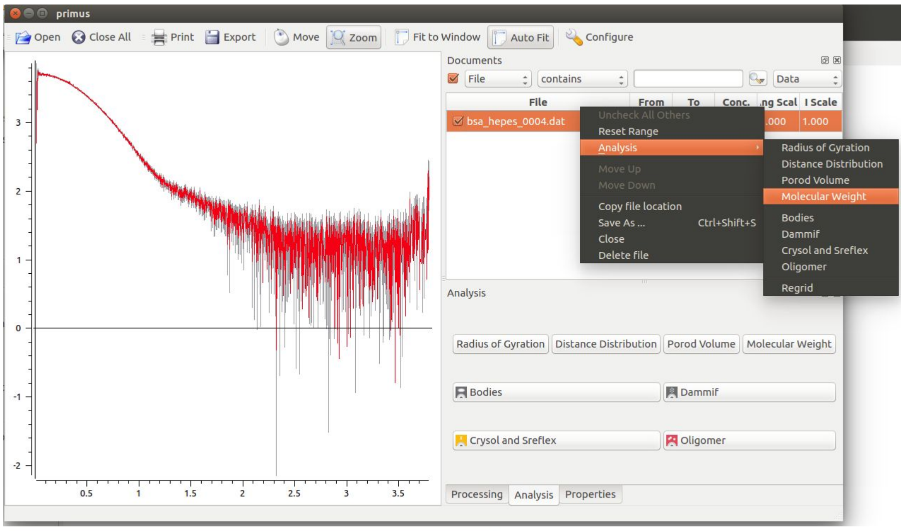

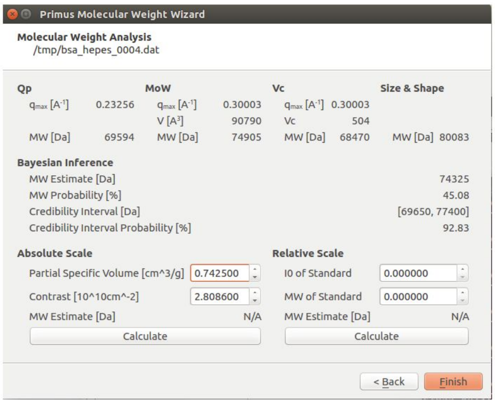

S7.2 Training data

The training data are contained in four different files, one for each method (mow.txt, qp.txt, vc.txt and sizeshape.txt) which are available following an ATSAS 2.8.3 download. The training data is built from the ideal scattering of all the unique PDBs, plus the same data at four noise levels (the highest one excluded). We have stored the training data in the form of a matrix, where the numbers represents the counts in each bin. The training data matrix is therefore a square matrix of length equal to the number of bins. The x-axis is the estimate from the methods (for instance, MM_{Qp}) and the y-axis is the actual MM (CRYSOL MM from the PDB) of the data. Finally, the bin edges are also available in the binedges.txt file.
